# Laplace Transform Fitting as a Tool To Uncover Distributions of Reverse Intersystem Crossing Rates in TADF Systems

**DOI:** 10.1021/acs.jpclett.2c01864

**Published:** 2022-07-26

**Authors:** Daniel Kelly, Larissa G. Franca, Kleitos Stavrou, Andrew Danos, Andrew P. Monkman

**Affiliations:** Department of Physics, Durham University, South Road, Durham DH1 3LE, United Kingdom

## Abstract

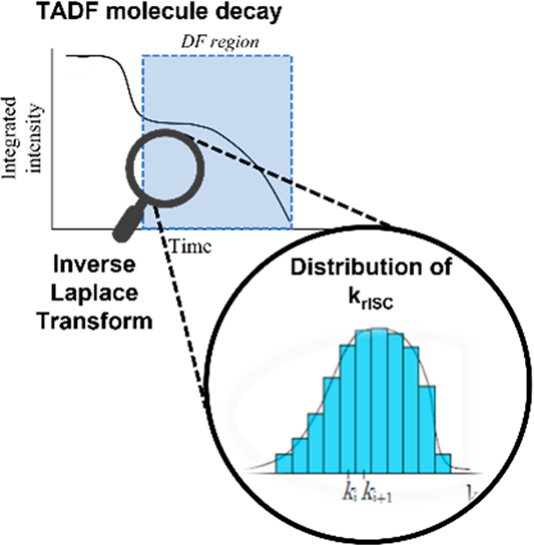

Donor–acceptor (D–A) thermally activated delayed fluorescence (TADF) molecules are exquisitely sensitive to D–A dihedral angle. Although commonly simplified to an average value, these D–A angles nonetheless exist as distributions across the individual molecules embedded in films. The presence of these angle distributions translates to distributions in the rates of reverse intersystem crossing (*k*_rISC_), observed as time dependent spectral shifts and multiexponential components in the emission decay, which are difficult to directly quantify. Here we apply inverse Laplace transform fitting of delayed fluorescence to directly reveal these distributions. Rather than a single average value, the crucial *k*_rISC_ rate is instead extracted as a density of rates. The modes and widths of these distributions vary with temperature, host environment, and intrinsic D–A torsional rigidity of different TADF molecules. This method gives new insights and deeper understanding of TADF host–guest interactions, as well as verifies future design strategies that target D–A bond rigidity.

Thermally activated delayed fluorescence (TADF) molecules have attracted tremendous interest in the field of organic light-emitting diodes (OLEDs).^1,2^ This is due to their ability to harvest triplet states without the use of expensive/rare heavy metals.^3^ A typical TADF molecule contains electron donor (D) and acceptor (A) units, usually linked by a C–N bond or in some cases bridged by a spiro carbon atom. The D–A structure enables a reduction of the energy gap between singlet and triplet states (Δ*E*_ST_) by minimizing the spatial overlap between the highest occupied and the lowest unoccupied molecular orbitals (HOMO and LUMO, respectively).^4^ Such an orbital pattern usually results in the formation of charge transfer (CT) excited states. Small Δ*E*_ST_ facilitates the conversion of triplet states into emissive singlet states via thermal activation. Therefore, minimizing Δ*E*_ST_ increases the efficiency of reverse intersystem crossing rates (*k*_rISC_), leading to an enhancement of the TADF efficiency. To allow a spin flip transition from triplet state to singlet state, a third mediating state such as locally excited triplet state (^3^LE) is required. The energy of this intermediary triplet state should be close to that of ^3^CT/^1^CT in order to allow for efficient vibronic coupling with the ^3^CT and for spin–orbit coupling with ^1^CT.^5^

The condition to minimize the electron exchange energy and thus Δ*E*_ST_ can be fulfilled by twisting the donor and the acceptor to near orthogonality. Spiro TADF molecules have their orthogonality enforced by the tetrahedral spiro carbon atom,^6^ whereas relative rotation between donor and acceptor is allowed in C–N linked molecules. In this case, a small variation in the D–A dihedral angle causes a distribution of Δ*E*_ST_ and *k*_rISC_ rates as a consequence.^7^ In solid state, the existence of this distribution is even more pronounced, as the host restricts motions on the guest molecules. This leads to the existence of dispersion of fixed D–A dihedral angles in the guest molecules, as observed in the time-resolved photoluminescence (TRPL) decay as a complex multiexponential decay of delayed fluorescence.^8,9^

Considerable work has been done to control D–A angles in TADF materials to maximize *k*_rISC_.^10^ For example, the attachment of heavy adamantyl groups or the linkage of a diphenyltriazine acceptor in carbazole donors was reported to restrict torsional motions.^11,12^ Introducing or relieving steric influences can also significantly impact the D–A angles and therefore also TADF performance.^7,13−15^

Beyond the synthetic approaches, the effect of variation in D–A dihedral angles of TADF emitters has been studied theoretically and experimentally by TRPL.^16−19^ There now exist many models to estimate the *k*_rISC_ from TRPL measurements, each with a unique mixture of strengths and limiting assumptions.^20^ Generally, these methodologies are described by the equilibrium model^21,22^ or estimated from a relationship between photoluminescence quantum yield and the lifetime of prompt (PF) and delayed fluorescence (DF).^23,24^ On the basis of that, Haase et al.^25^ proposed a simplified kinetic model to extract the rate constants of TADF from TRPL, which was shown to give reliable results. These existing methods share the same core assumption of a singular *k*_rISC_ rate. Therefore, they are unable to consider the distribution of *k*_rISC_ rates that occur in film samples, returning only a representative average value, while a methodology that considers the existence of the *k*_rISC_ rate distribution in TADF systems remains unexplored. Methods using more sophisticated approaches such as machine learning have been recently applied to TRPL data in the context of nanocrystals and photovoltaic materials.^26,27^

Herein, we propose a different and simple approach, which uses inverse Laplace transform fitting of the DF decay to extract a distribution of *k*_rISC_ rates. By using this methodology, we are able to analyze the influence of host and temperature on the distribution of decay rates of TADF molecules. More importantly, this method can provide useful data for theoretical modeling of *k*_rISC_ rates in a variety of TADF systems.

An intensity decay curve arising from the sum of a statical distribution of *N* single exponential decays (each with decay rate *k*_*n*_ and initial amplitude *a*_*n*_) can be represented as
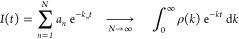
1where each summed exponential term represents the DF decay of subsets of TADF molecules in the film with a specific D–A angle and therefore a specific Δ*E*_ST_ and *k*_rISC_ rate. In the limit of continuous *k*, this form coincides with the Laplace transform of a rate distribution function, ρ(*k*). Since the intensity profile  is obtained directly from TRPL data, the distribution of rates that generate the DF decay can thus be extracted by computing the inverse Laplace transform.

Inverse Laplace transform fitting is challenging to perform for a typical TRPL data set, since only the real part of the transform is available for sampling and the data density may not be high enough for the available numerical algorithms.^28^ To facilitate computation, the integral (eq 1) was approximated as a Riemann sum (eq 2), with the range of *k* values of experimental interest split into *N* bins of width . Because of the large range of *k* values of interest, these bins were evenly distributed across the base 10 logarithm of *k*. This discretization reverts the integral to a similar form as the original sum of exponentials while still giving access to the (now also discretized) rate distribution function :
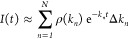
2

To practically perform the fitting, the discretized  was initialized as a sequence of *N* independent variables. The sum for  could then be computed and was passed to a least-squares regression algorithm using the scipy.optimize package in Python 3.6 to obtain the set of  that gave the best fit to the normalized experimental TRPL DF data. The optimized discrete values of  plotted against  form the distribution of rates for a particular TRPL data set. This process is summarized graphically in Scheme 1.

**Scheme 1:**
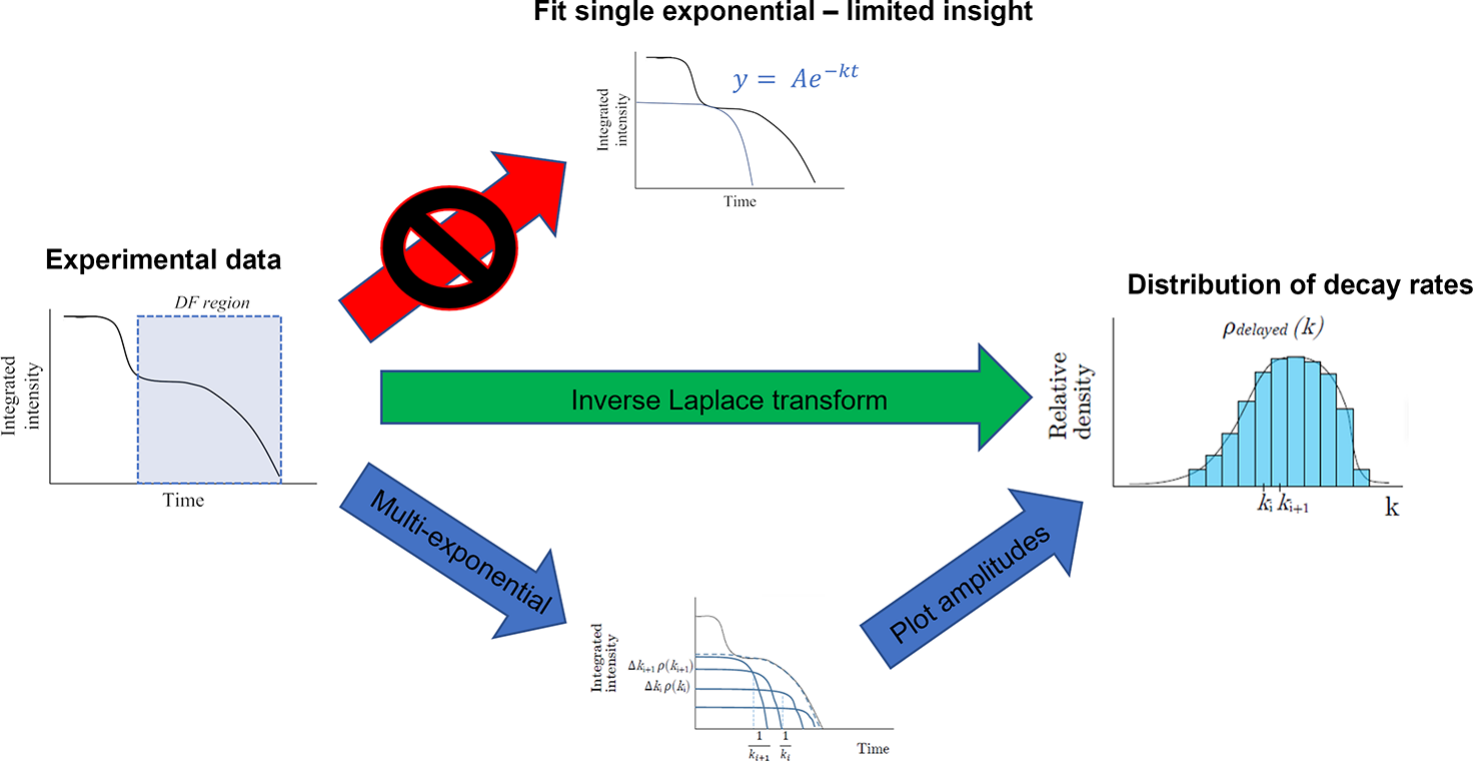
Extraction of Decay Rate Distributions from TRPL Data Using Inverse Laplace Transform Fitting

Furthermore, to increase the data resolution of the estimated ρ(*k*), i.e., to make the estimation more continuous-like, the process was repeated for several *k* values differing by a small amount, all inside the range of a singular bin. Since for a given TRPL DF data set the maximum attainable *N* was determined by input data density (above  the output was visibly overparameterized), repeating the calculation for different *k* does not increase accuracy but allows a better extraction of  as a continuous curve approximation.

To assess the validity of the optimization-based inverse Laplace transform, we first applied it in the kinetic decays as a function of temperature, commonly used as the measurement to probe the TADF mechanism. Figure 1a shows the distributions of *k*_rISC_ rates for solid films of the well-studied emitter **DDMA-TXO2**(7,29−31) in UGH host (10 wt % loading) at different temperatures. With decreasing temperature, the distribution broadens toward slower decay rates. This is a result of molecules experiencing slower *k*_rISC_ as the lower temperature reduces the rate of thermal activation. Interestingly, while the bulk of the distribution shifts to lower rates, we note that the high-rate onset remains invariant until 240 K and the peak of the distribution barely shifts. This indicates that a significant subset of the molecules reach their maximum potential *k*_rISC_ rate at temperatures as low as 240 K, i.e., saturation, with increased temperature enhancing the *k*_rISC_ rate of the slower side of the distribution (left side) but having no further enhancement effect on the fast side (right side). Below ∼240 K, the peak of the rate distribution begins to shift to slower values, while the amplitudes of the fastest rates (∼10^5^s^−1^ at RT) decrease in relative density. We suggest that a phosphorescence contribution becomes significant at these temperatures despite not being conclusively resolvable in individual spectra, although the significant broadening of the distribution likely still includes some longer lived DF contribution. Through this motional hindrance, molecular conformations with stronger LE character, which could not be observed at room temperature, can now be observed as slow *k*_rISC_ rate contributors with long DF lifetimes.

**Figure 1:**
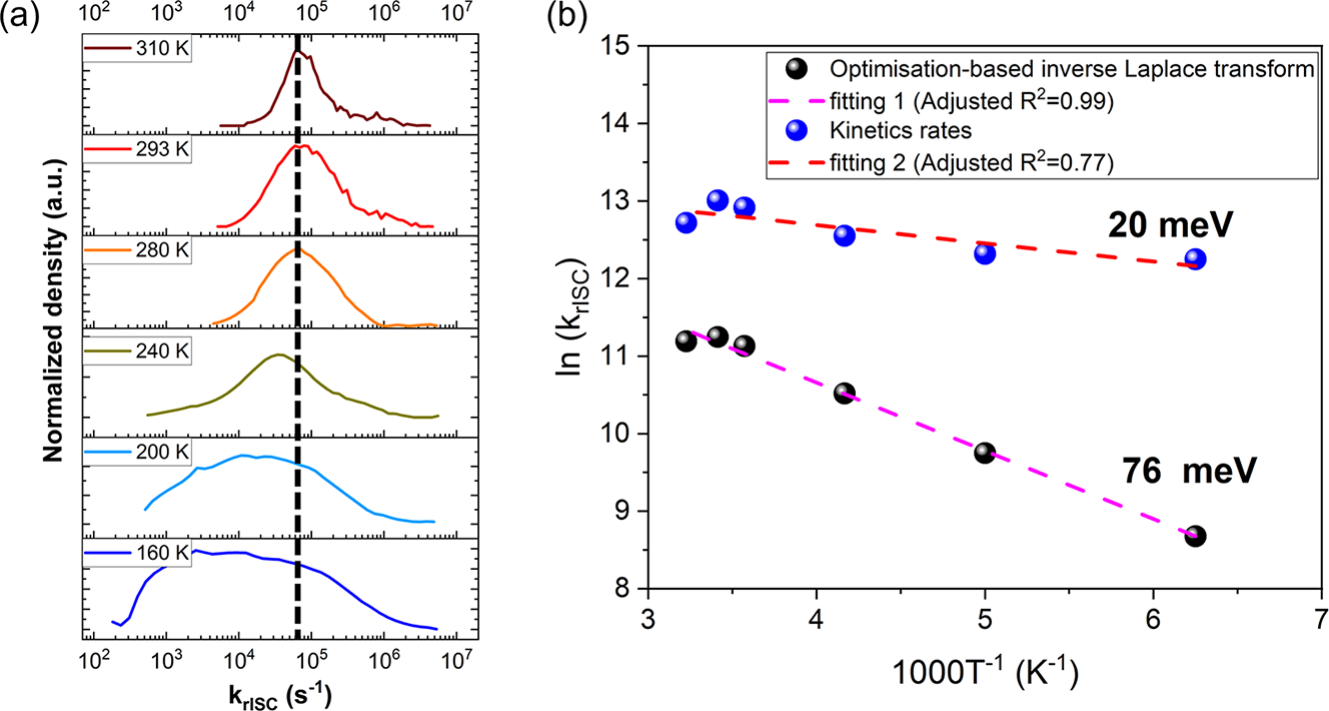
(a) Distributions of *k*_rISC_ rates of **DDMA-TXO2**:UGH solid film at different temperatures. TRPL data are shown in Figure S2. (b) Temperature dependence of the DF rate in an Arrhenius plot of **DDMA-TXO2**:UGH solid film.

To further analyze the rate distribution plots, a log-normal Gaussian curve was used to approximate the peak and numerically extract the rates corresponding to the peak centers (i.e., the distribution mode). From these values, we obtained the Arrhenius plot, as shown in Figure 1b. The linear dependence of *k*_rISC_ with the inverse of temperature is clearly observed, according to eq 3.
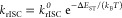
3

From this Arrhenius plot, Δ*E*_ST_ is estimated to be 76 ± 3 meV, while the rates from kinetic fitting of the same decays instead yield Δ*E*_ST_ of 20 ± 5 meV.^25^ Comparing both approaches, the optimization-based inverse Laplace transform gives a better linear relationship and a larger value of Δ*E*_ST_, much closer to the experimental value of 92 ± 5 meV (Figure S3) from optical spectra. These different Δ*E*_ST_ values are likely related to the region of fitting, with the Laplace fit better representing the entire DF region and hence its distribution giving a Δ*E*_ST_ with better weighting to slower rates contributions. In contrast, the kinetic rates fitting takes into account mainly the initial (fastest) part of the DF regime (Figure S4), where the faster *k*_rISC_/smaller Δ*E*_ST_ molecules are dominant. The inverse Laplace transform considers a range about the most probable *k*_rISC_ rates, giving the Δ*E*_ST_ associated with it.

Extending beyond temperature, we applied the method to decays of **DMAC-TRZ** in solutions (0.8 mM concentration) as well as doped (1 wt % loading) in a range of five host matrices, which present different dielectric constants and rigidities. In solution very narrow rate distributions were obtained in all cases, as expected when molecules can dynamically reorganize during the decay, emitting predominantly as they sample their lowest-Δ*E*_ST_ fastest *k*_rISC_ geometries. Increasing the polarity of the solvent, we observed a shift of the peak distribution toward faster *k*_rISC_ rates (Figure 2a) as ^1^CT is stabilized and Δ*E*_ST_ decreases. This is due to the formation of excited states with CT character in TADF molecules. Therefore, increasing polarity of the solvent decreases the energy of the ^1^CT/^3^CT manifolds, bringing them closer to the ^3^LE, which remains unaffected. Often the ^1^CT–^3^LE energy gap is equal to Δ*E*_ST_; hence stabilizing the CT leads to a reduction in the Δ*E*_ST_.

**Figure 2:**
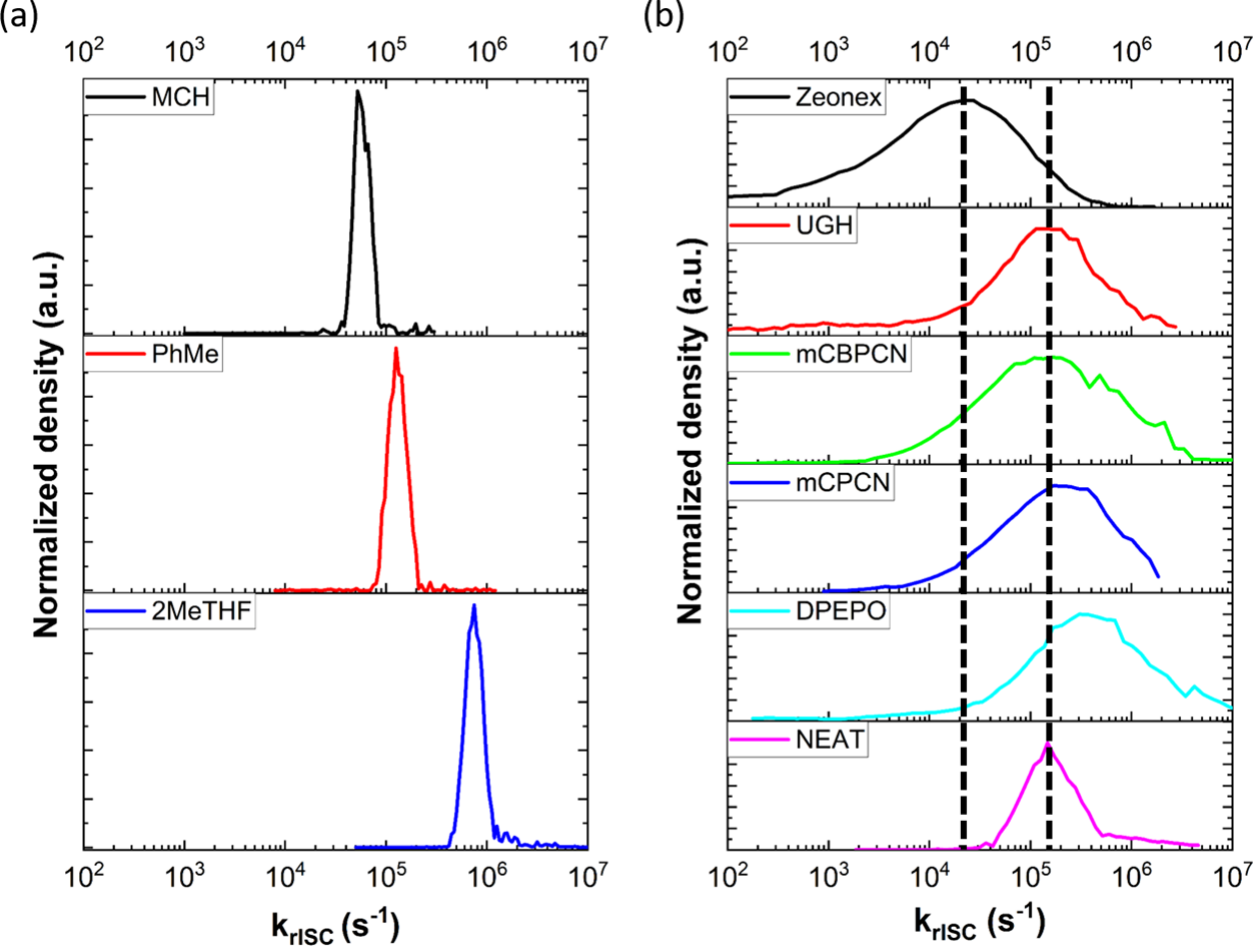
Distribution of *k*_rISC_ rates of (a) **DMAC-TRZ** in solvents of increasing polarity at 0.8 mM concentration and (b) 1% loading drop casted films of **DMAC-TRZ** in different hosts. All measurements were performed at room temperature. TRPL decay can be found in Figure S5.

While the solution distributions were therefore entirely as expected, the solid-state results provide a more complex picture (Figure 2b). Due to packing effects that hinder molecular motions, these molecules become pinned in their as-deposited molecular configurations with limited ability to sample other geometries. As shown by Dhali et al.,^32^ the **DMAC-TRZ** ground state equilibrium D–A dihedral angle is ∼90°, while vibration/twisting modes in the excited state geometry lead to a dynamic range of angles of ≤90°. While in solution, a slightly polar solvent can contribute to a fast stabilization of the CT state, as seen in the case of toluene; in solid-state the environmental response is very different because the host molecules cannot realign to relax the change in (the TADF) molecular dipole moment on photoexciation. The latter, along with the packing properties due to solid-state guest–host interactions, results in a distribution of destabilized or multienergetically “stabilized” molecular conformations. As a result, a broader distribution is observed in all solid-state hosts.

In the zeonex polymer matrix, a very broad distribution of rates is observed. This host also gives the smallest peak *k*_rISC_ value because of the low host dielectric constant, resulting in a large Δ*E*_ST_ for **DMAC-TRZ** and so low average *k*_rISC_ rate. The low rigidity of the polymer itself is still enough to restrict reorganization of **DMAC-TRZ** emitter, resulting in the broad distribution that spans from ∼10^3^ up to ∼10^5^ s^–1^. In small molecule hosts, the enhanced rigidity and tight packing of the environment give higher *k*_rISC_ peak values in all cases; DPEPO gives slightly higher performance, while UGH the lowest, similar to what we have previously reported (Table S1).^9^ Within these small molecule hosts, neutral UGH presented a slightly narrower distribution while mCBPCN the broadest, on a logarithmic scale. Because all of these hosts are expected to be similarly rigid, these differing distribution widths suggest that they permit different ranges of D–A angles to be “locked in” to the TADF molecules during the deposition process. Minimising this inhomogeneity is highly advantageous in devices, and so engineering new hosts to minimize inhomogeneous dihedral angles, guided by the study of decay rate distributions, could be extremely fruitful for device color purity and performance.

Although many parameters may affect the distribution width, when comparing **DMAC-TRZ** in small molecule hosts and neat film results, it is clear that in the latter case there is a narrow dispersion, compared to all guest–host films but slightly wider compared to solution due to intermolecular interaction. This suggests that the different physical properties of these small molecule hosts along with the guest–host interactions directly affect the guest’s excited-state conformations and thus *k*_rISC_ rates.

Moving beyond hosts, in Figure 3 distributions are compared between **DMAC-TRZ** and **ACRSA** molecules (in both UGH and DPEPO matrices, which have similar rigidity but different dielectric constants). These emitters were chosen to showcase the effect of different D–A linkages; while **DMAC-TRZ** has a flexible C–N bridging bond, **ACRSA** contains a rigid spiro carbon atom that hinders almost all motion between D and A.^33^ As a consequence, **ACRSA** molecules all have near-identical molecular geometry in film and a very narrow distribution of *k*_rISC_ rates that is nearly identical in both hosts. Interestingly, the peak of the distribution is also unchanged for **ACRSA** in the two different hosts (Figure S6), confirming the recently reported invariance it possesses to host environment,^33^ in absolute contrast to **DMAC-TRZ**.

**Figure 3:**
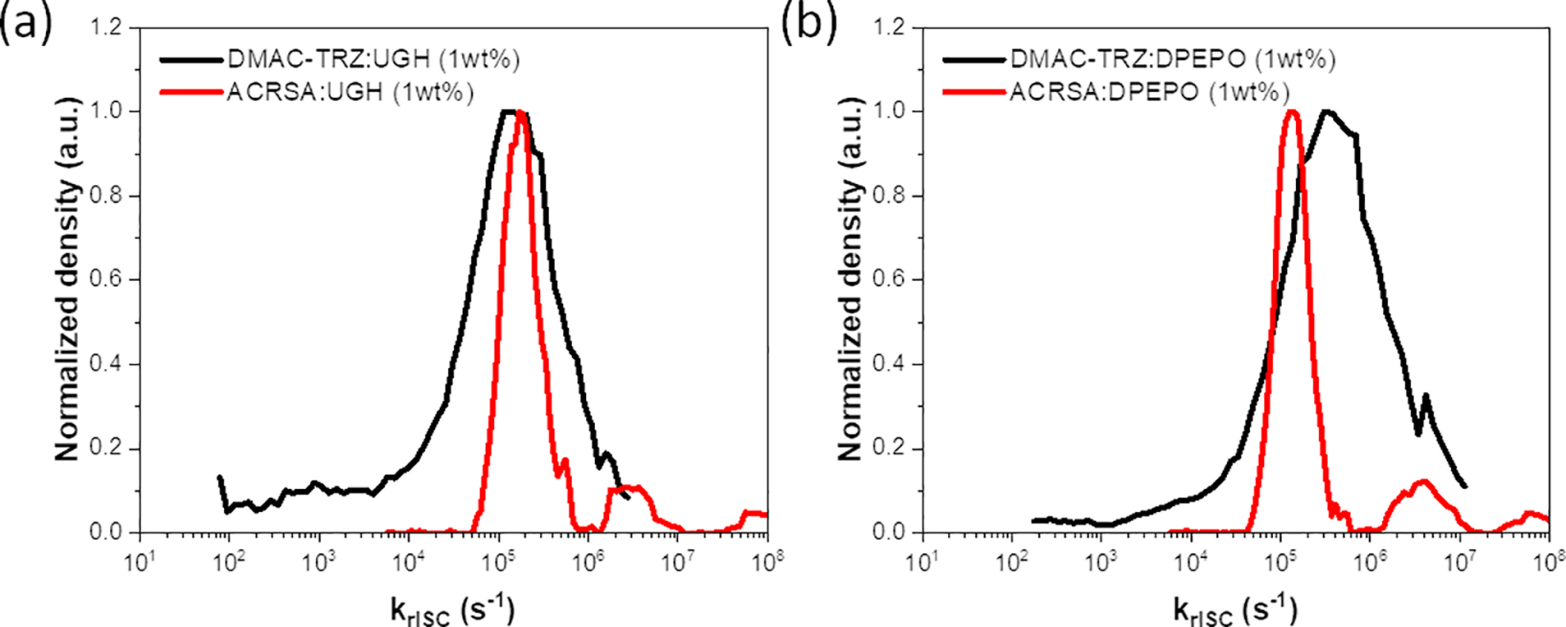
Comparison of the distribution of *k*_rISC_ rates between **DMAC-TRZ** and **ACRSA** in (a) UGH and (b) DPEPO, as a host matrix at 1% concentration. TRPL decay can be found in Figure S7.

We also applied the inverse Laplace fitting method to the DF of a set of recently reported **DMAC-BZN** isomers, which were calculated to have different D–A bond rigidity^14^ due to resonance and intramolecular dipole interactions. In that work, calculated potential energy surfaces indicated that D–A dihedral rocking should be more relaxed in the *meta* and *para* isomers. This would then lead to a wider range of D–A bond geometries present in the as-deposited films, which we see subtly reflected in the widths of the fitted distributions (Figure S8). This effect is small, which we suggest is due to the rigidity of the DPEPO host in the measurements, not reflected in the gas-phase calculations of potential energy surfaces. The modes of these distributions also conform to the expected order of *k*_rISC_ rates in these materials, as determined by kinetic fitting (Table S2).

In summary, we have developed a new approach to fitting TADF emission decays, which gives direct information on the effect of D–A dihedral angle distributions present in films by using an inverse Laplace transform. Our results reveal insight that is inaccessible to single-value fitting approaches: for molecules with small Δ*E*_ST_, decreasing the temperature broadens the distribution of *k*_rISC_ rates but does not immediately shift the mode or the fastest contributors. Very narrow rate distributions are observed in solution because of the allowed molecular motions/relaxation, while in solid-state, the rigidity of the environment hinders those processes and the distribution can be extremely broad, explaining the presence of long-lived DF in solid hosts compared to solution with immediate implications for EQE roll-off in OLEDs. Finally, comparing two molecules with different intrinsic dihedral rigidity, we recover a much narrower distribution for the rigid material, while the method is also able to resolve much subtler differences in molecular rigidity that were previously only accessible in calculations. Inaccessible to previous single-rate methods, we propose that this method will allow researchers to quantify the subtle effects of both host and intrinsic molecular rigidity and independently verify future design strategies for TADF materials.

## Supporting Information Available

General experimental procedures and time-resolved photoluminescence decay (PDF)
