# Supplementary material for: Laplace Transform Fitting as a Tool To Uncover Distributions of Reverse Intersystem Crossing Rates in TADF Systems
Source: J Phys Chem Lett. 2022 Jul 26;13(30):6981–6. doi: 10.1021/acs.jpclett.2c01864 (PMC9358706; doi:10.1021/acs.jpclett.2c01864)
Supplement: Supplementary file 1 — jz2c01864_si_001.pdf [file jz2c01864_si_001.pdf]

Supporting Information

Laplace transform fitting as a tool to uncover distributions of reverse intersystem crossing rates in TADF systems

Daniel Kelly,[a] Larissa G. Franca,[a] Kleitos Stavrou,[a] Andrew Danos,[a] Andrew P. Monkman[a,]*

[a] Department of Physics, Durham University, South Road, Durham, DH1 3LE, United Kingdom.

AUTHOR INFORMATION

Corresponding Author

*Andrew P. Monkman: a.p.monkman@durham.ac.uk

Experimental section

1. Sample preparation

DDMA-TXO2:UGH films were made by coevaporation deposition at an evaporation rate of 0.9 Å/s for UGH host molecule and 0.1 Å/s for **DDMA-TXO2** molecule. The evaporated films were prepared using a Kurt J. Lesker Super-Spectros 200 deposition system under vacuum, 10^{-7} mbar, and a final thickness of 100 nm was obtained. Degassed solutions of **DMAC-TRZ** were prepared at 0.8 mM concentration in methylcyclohexane (MCH), toluene (PhMe), and 2-methyl tetrahydrofuran (2MeTHF). Degassed solutions were obtained by 5 freeze-pump-thaw cycles to remove all dissolved oxygen. Solid state samples were fabricated by drop casting onto quartz. **DMAC-TRZ** drop casted films were produced at 1% w/w in host matrix (Zeonex, DPEPO (bis[2-(diphenylphosphino)phenyl]ether oxide),), UGH (m-bis(triphenylsilyl)benzene), mCPCN ([9-(3-(9H-carbazol-9-yl)phenyl)-9Hcarbazole-3-

carbonitrile) and mCBPCN (9-(30-(9H-Carbazole-9-yl)-5-cyano[1,10-biphenyl]-3-yl)-9H-carbazole-3-carbonitrile)). For neat **DMAC-TRZ** drop casted film, solution of 1 mg/mL were dropped onto substrate. **ACRSA** drop casted films were produced at 1% w/w in UGH and DPEPO. **DMAC-BZN** isomers films were produced by drop casting at 10% w/w in DPEPO host.

2. Photophysical characterization

Time-resolved measurements were detected by a spectrograph and a gated iCCD camera (Stanford Computer Optics), where samples were excited with a Nd:YAG laser (EKSPLA), 10 Hz, 355 nm.

Chemical structures:

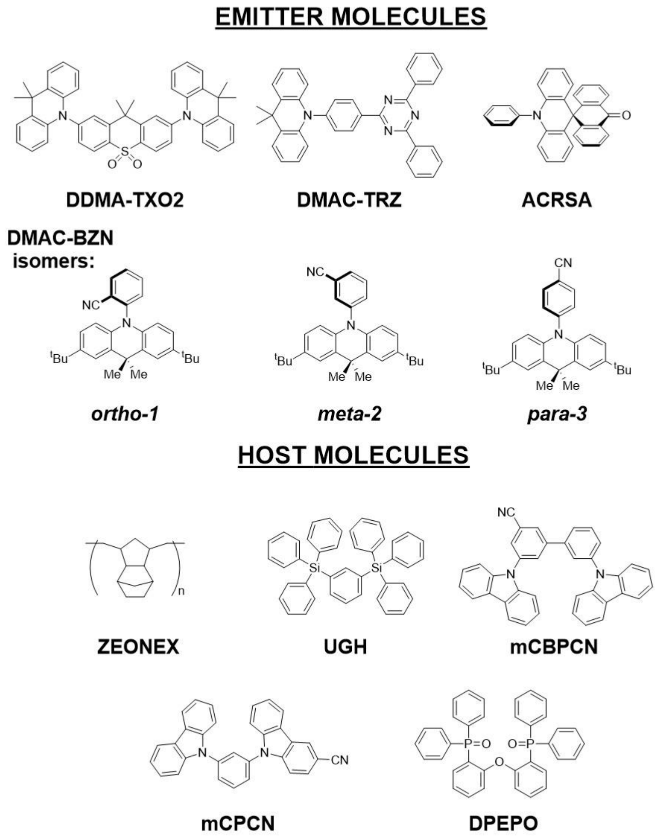

Figure S1: Chemical structures of the emitter molecules and host molecules used in this work.

Photophysical properties:

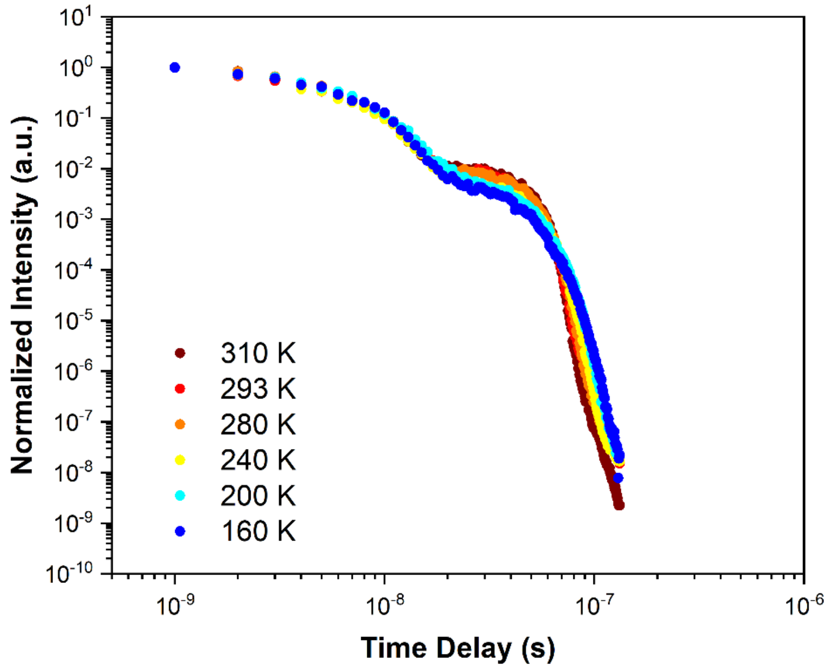

Figure S2: TRPL data of **DDMA-TXO2:**UGH solid film at different temperatures.

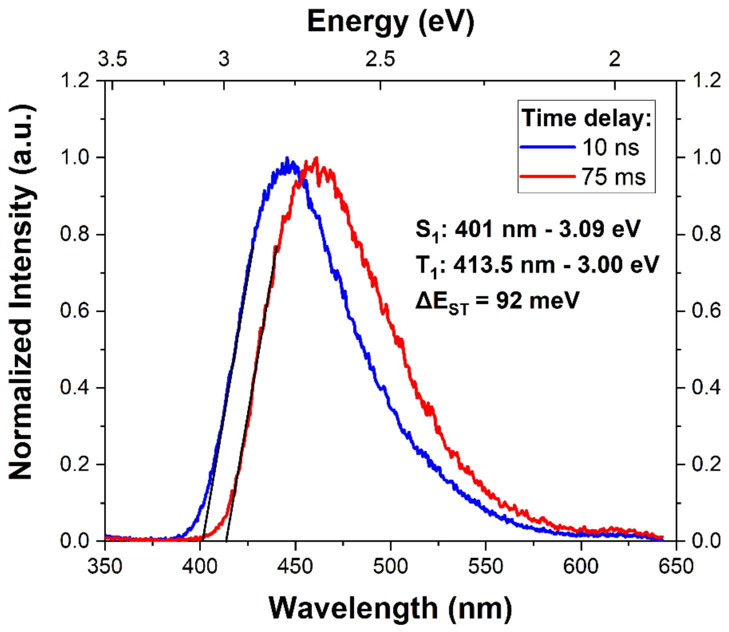

Figure S3: Prompt fluorescence (blue curve) and phosphorescence (red curve) spectra of **DDMA-TXO2**:UGH recorded at room temperature and 20 K, respectively. Note that the prompt spectrum was recorded with 10 ns time delay, while the phosphorescence spectrum was at 75 ms.

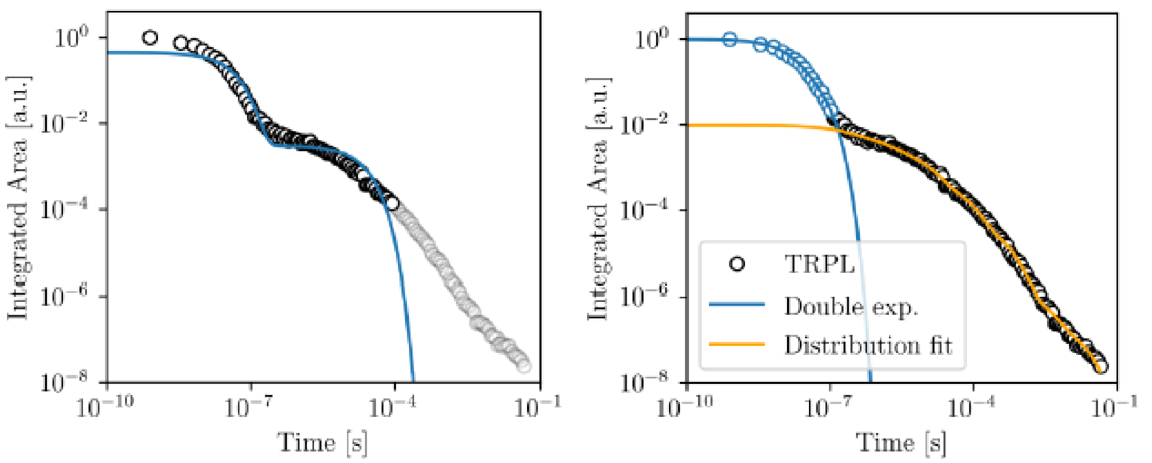

Figure S4: TRPL data of **DDMA-TXO2** in UGH host at 200 K. Model fits provided using: on the left, by the known kinetic fit; on the right, by optimisation-based inverse Laplace transform.

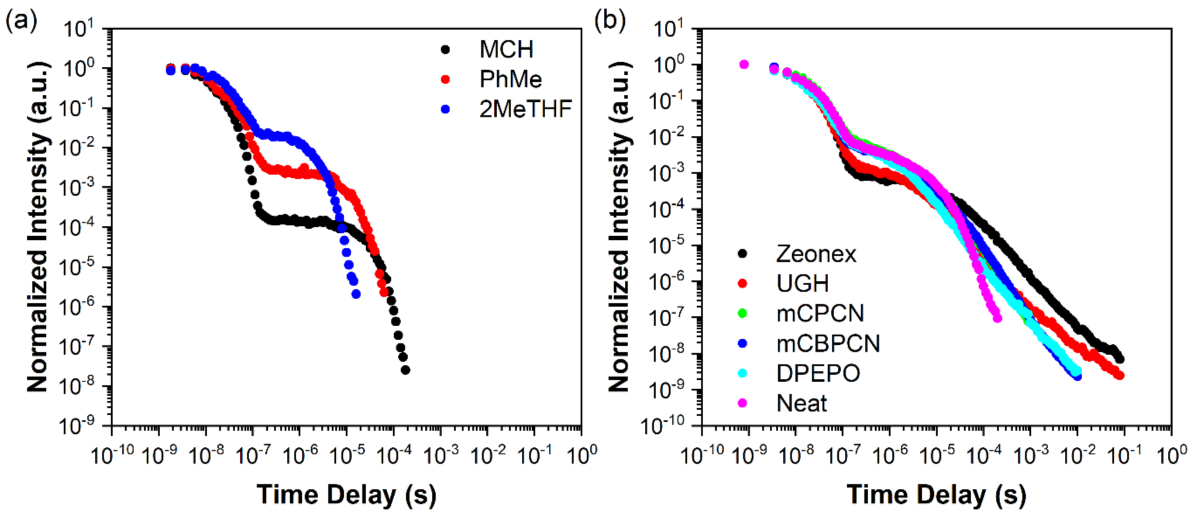

Figure S5: TRPL data of (a) **DMAC-TRZ** in solvents of increasing polarity at 0.8 mM concentration and (b) 1% loading drop casted films of **DMAC-TRZ** in different hosts

Table S1: Comparison of the rISC rates obtained by the known kinetic fit and optimisation-based inverse Laplace transform. [a]Data obtained from literature.[1]

**DMAC-TRZ 1% w/w in Host**	**Zeonex**	**UGH**	**mCBPCN**	**mCPCN**	**DPEPO**	**Neat Film**
[a]$\mathbf{k_{rISC}}$ **(s^{-1})**<br>(Kinetic fit)	1.7 x10^5	4.8 x10^5	9.3 x10^5	9.6 x10^5	1.1 x10^6	8.1 x10^5
$\mathbf{k_{rISC}}$ **(s^{-1})**<br>(Laplace distribution)	2.1x10^4	1.5x10^5	1.4x10^5	2.0x10^5	3.7x10^5	1.5x10^5

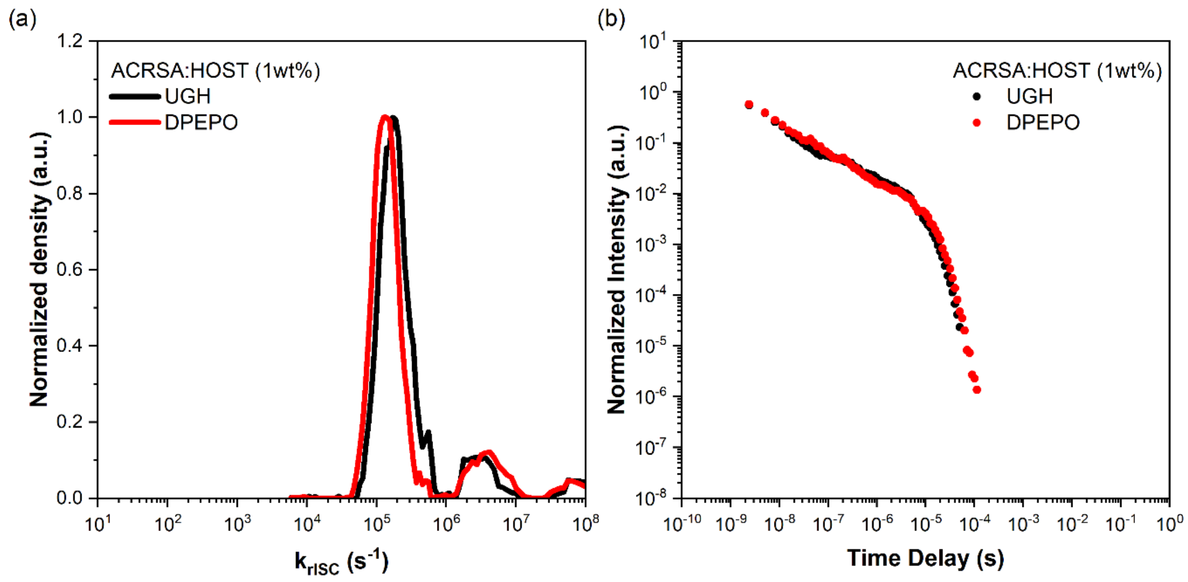

Figure S6: Distribution of rISC rates of (a) 1% loading drop casted films of **ACRSA** in UGH and DPEPO. TRPL data are shown in (b).

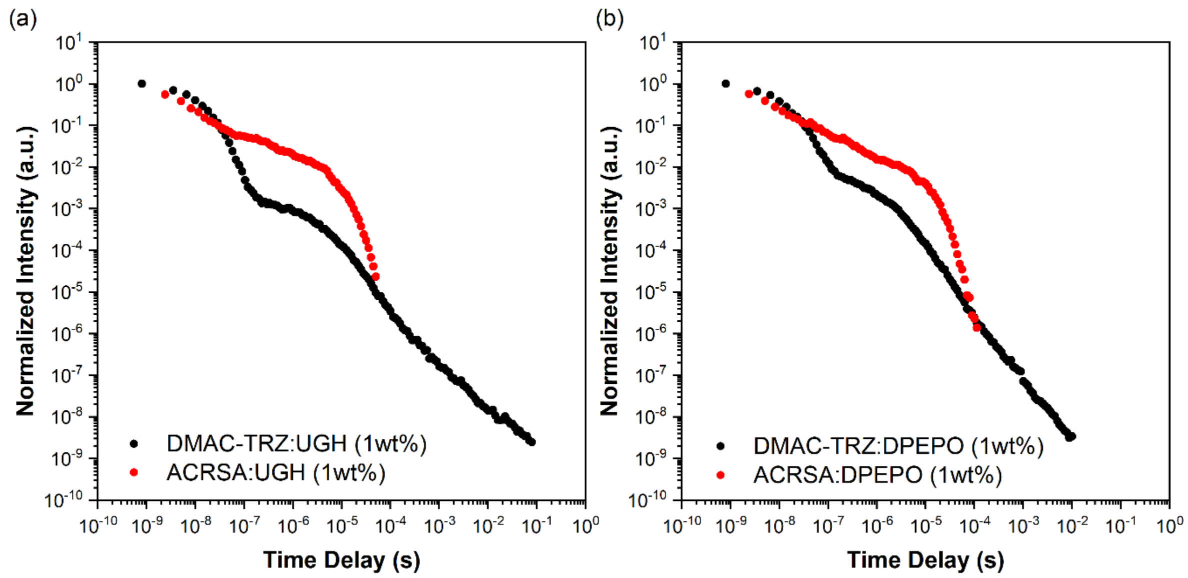

Figure S7: TRPL data of **DMAC-TRZ** and **ACRSA** in (a) UGH and (b) DPEPO, as a host matrix at 1% concentration.

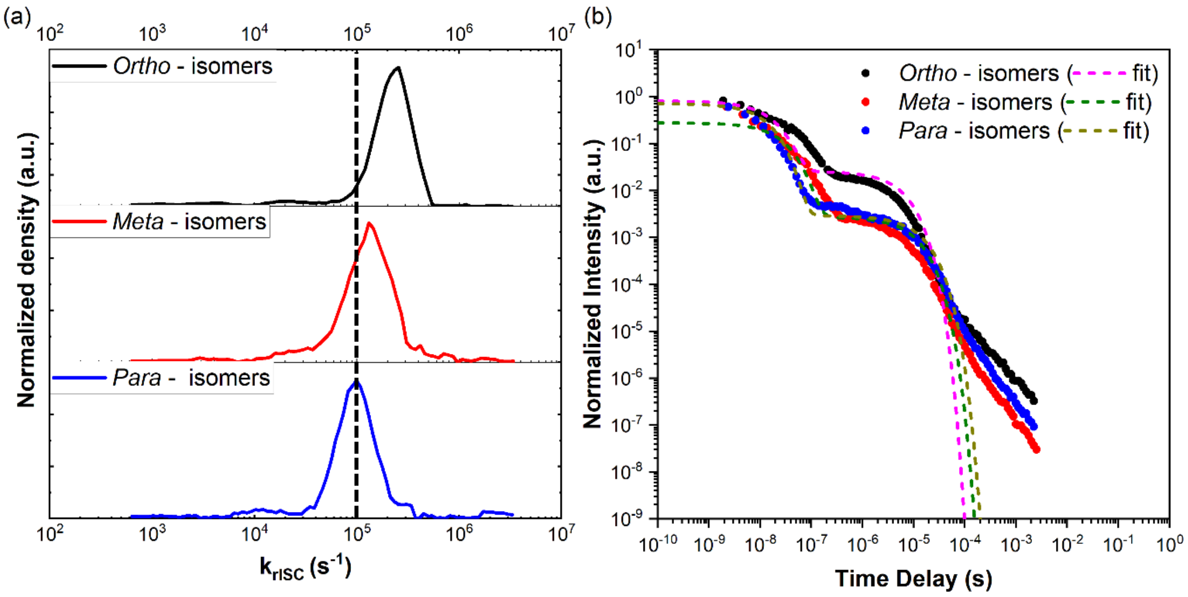

Figure S8: Distribution of rISC rates of (a) **DMAC-BZN** isomers in DPEPO, as a host matrix at 10% concentration. TRPL data and fitting are shown in (b).

Table S2: Comparison of the rISC rates obtained by the known kinetic fit and optimisation-based inverse Laplace transform of TRPL data in **Figure S8**.

**DMAC-BZN 10% w/w in Host**	**Ortho**	**Meta**	**Para**
[a]**k_{rISC} (s^{-1})** (Kinetic fitting)	1.9 ×10^6	4.3 ×10^5	3.6 ×10^5
**k_{rISC} (s^{-1})** (Laplace distribution)	2.3×10^5	1.4×10^5	9.7×10^4

References:

(1) Stavrou, K.; Franca, L. G.; Monkman, A. P. Photophysics of TADF Guest–Host Systems: Introducing the Idea of Hosting Potential. *ACS Appl. Electron. Mater.* **2020**, *2* (9), 2868–2881. https://doi.org/10.1021/acsaelm.0c00514.
